# Primary salivary gland-type polymorphous adenocarcinoma in the lung: A case report and literature review

**DOI:** 10.1097/MD.0000000000029224

**Published:** 2022-05-13

**Authors:** Hong-Bo Xu, Mai-Qing Yang, Jing-Ru Wang, Hong-Feng Qi, Xu-Yong Lin, Hai-Ning Zhang, Hong-Tao Xu

**Affiliations:** a Department of Blood Transfusion, Changyi People's Hospital, Changyi, China; b Department of Pathology, Weifang People's Hospital (First Affiliated Hospital of Weifang Medical University), Weifang, China; c Department of Clinical Pathology, School of Clinical Medicine, Weifang Medical University, Weifang, China; d Department of Thoracic and Cardiac Surgery, Changyi People's Hospital, Changyi, China; e Department of Pathology, The First Affiliated Hospital and College of Basic Medical Sciences, China Medical University, Shenyang, China.

**Keywords:** lung neoplasm, polymorphous adenocarcinoma, polymorphous low-grade adenocarcinoma, salivary gland-type tumor

## Abstract

**Rationale:**

Polymorphous low-grade adenocarcinoma is a low-risk infiltrative malignant tumor of the salivary glands. However, some of these tumors are more malignant than the low-grade tumors and therefore, according to the most recent recommendation of the World Health Organization, they are renamed as polymorphous adenocarcinomas (PACs). Primary polymorphous low-grade adenocarcinomas/PACs of the lungs are rare. Herein, we report a case of primary PAC of the lung with bronchial cartilage and perineural invasion, and lymph node metastasis.

**Patient concerns:**

A 58-year-old man had developed fever half a month prior, without chills or other accompanying symptoms, and the underlying reasons were unknown. His self-measured temperature was up to 39°C, accompanied by cough and expectoration, yellow and thin sputum, and shortness of breath. The patient's general state was normal, and respiratory sounds originating from the right lung were weak. Enhancement computed tomography revealed that the bronchial lumen of the basal segment of the lower lobe of the right lung was narrow; soft tissue density nodules were seen, with a range of approximately 2.4 cm × 1.3 cm.

**Diagnosis:**

Based on clinical information, morphological features, and immunohistochemistry results, the pathological diagnosis was primary PAC of the lungs.

**Intervention:**

Thoracoscopic resection of the middle and lower lobes of the right lung was performed, further extended dissection of the mediastinal lymph nodes was performed.

**Outcomes:**

The postoperative course was uneventful.

**Lessons:**

Primary PAC of the lung is rare and may cause misdiagnosis. When encountering a lung tumor with diverse tissue structures, uniform cell type and nerve invasion, we should consider the possibility of PAC. Morphological and immunohistochemical features can be useful for diagnosing primary PAC of the lungs.

## 1 Introduction

Polymorphous adenocarcinoma (PAC), previously known as polymorphous low-grade adenocarcinoma (PLGA), is the second most common minor salivary gland tumor of the oral cavity.^[1,2]^ PLGA is a low-grade malignant infiltrative tumor with low metastasis and excellent survival.^[3]^ However, a recent update made in the salivary gland section of the World Health Organization omitted the term “low-grade” because the data showed recurrence rates of the tumor in the minor salivary gland of head and neck up to 19%, and some cases transformed to high-grade malignancies.^[1,4,5]^

Metastasis of PAC from the head and neck to the lung is rare. Primary PACs in the lungs are extremely rare. These tumors are thought to arise from mucous glands that line the bronchial tree. Here, we report a case of high-grade PAC originating from the lung with bronchial cartilage and perineural invasion and lymph node metastasis, without a known history of primary salivary gland tumor.

## 2 Case presentation

### 2.1 Ethic approval

This study was approved by the China Medical University Institutional Review Board for Human Studies. The ethical board approval number is LS[2021]009. Written informed consent was obtained from the patient for the publication of this case report and accompanying images. This study was performed in accordance with the Declaration of Helsinki.

### 2.2 Clinical history

**Figure 1:**
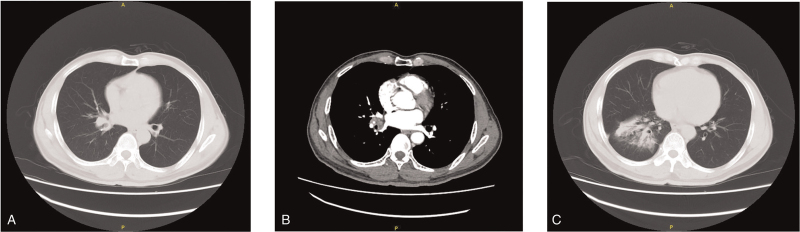
Computed tomography of the chest. A and B, computed tomography scan showing the bronchial lumen of the basal segment of the lower lobe of the right lung, which is narrow; soft tissue density nodules are seen around the same, with a range of approximately 2.4 × 1.3 cm. C, Multiple solid shadows are observed in the distal lung field.

A 58-year-old man was admitted to our hospital for further treatment as he had been affected by fever for half a month and cough for 1 week. The patient had developed fever half a month prior, without chills or other accompanying symptoms, and the underlying reasons were unknown. The patient complained of fever symptoms more frequently than 1 week ago. His self-measured temperature was up to 39°C, accompanied by cough and expectoration, yellow and thin sputum, and shortness of breath. He was administered roxithromycin and antipyretic drugs, and the effect was poor. The patient's general state was normal, and respiratory sounds originating from the right lung were weak. Enhancement computed tomography (CT) revealed that the bronchial lumen of the basal segment of the lower lobe of the right lung was narrow; soft tissue density nodules were seen, with a range of approximately 2.4 × 1.3 cm. The plain CT value was 49 HU, which was increased after enhancement (CT value, 73 HU). Multiple solid shadows were observed in the distal lung field, and were significantly increased after enhancement. The right hilar lymph node was enlarged. The mediastinum was in the middle, and no enlarged lymph nodes were observed (Fig. 1). The preoperative diagnosis was right lower lobe lung cancer and severe community-acquired pneumonia. Thoracoscopic resection of the middle and lower lobes of the right lung was subsequently performed. During surgery, the resected lung specimen was collected to prepare frozen sections and pathological evaluation. The diagnosis was “malignant tumors, depending on routine paraffin sections and immunohistochemical identification and classification after the operation”. Subsequently, further extended dissection of the mediastinal lymph nodes was performed. The patient did not receive postoperative radiotherapy or chemotherapy and recovered well after surgery. Follow-up at 3 months did not reveal any evidence of recurrence or other metastatic diseases.

### 2.3 Immunohistochemical staining

The resected specimens were fixed with 10% neutral-buffered formalin, embedded in paraffin blocks, and cut into 4 μm thick sections. The sections were then stained with hematoxylin and eosin for histological assessment. Some tumor sections were immunostained with ready-to-use primary antibodies against broad-spectrum cytokeratin (CK), vimentin, CD117, CK5/6, CK7, P40, P63, thyroid transcription factor-1 (TTF-1), smooth muscle actin, CD56, napsin A, synaptophysin, S-100, and Ki-67 (Maixin, Fuzhou, China). Subsequent detection was performed using the streptavidin-peroxidase method after incubation with the primary antibody. Positive and negative controls were used appropriately.

### 2.4 Morphological and immunohistochemical findings

Grossly, the resected part of the lung specimen was 18 × 7 × 5 cm in size, and was cut along the bronchus. A fine circumscribed papillary tumor with a maximum diameter of 2 cm was observed 1.2 cm from the broken end of the bronchus. Most of the tissue was brittle and contained fish-like structures. Gray and pale areas were observed in the cut sections. The tumor mass was circumscribed, and the resection margins were free of the tumor. Eighteen tracheobronchial and subcarinal lymph nodes were dissected.

**Figure 2:**
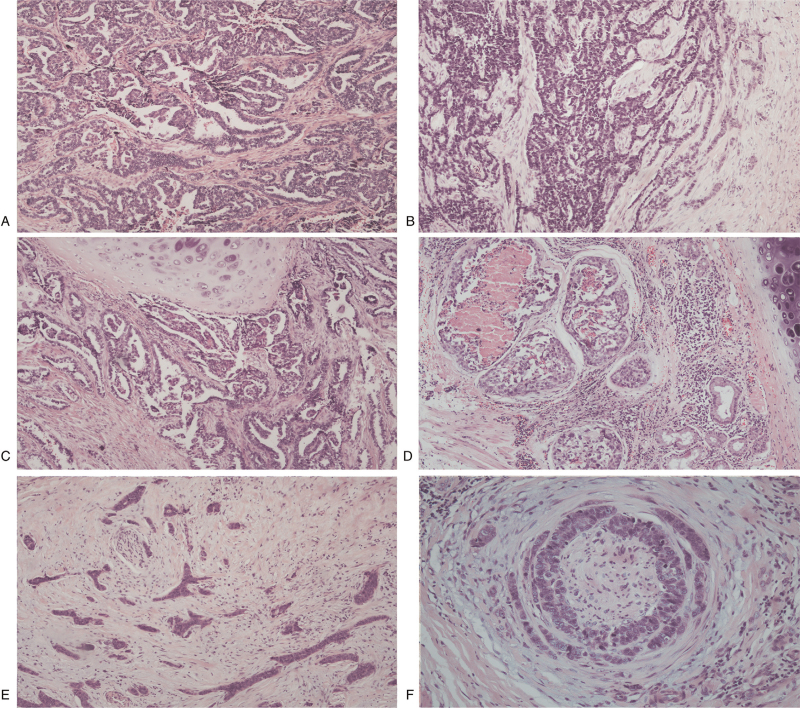
Histological features associated with the polymorphous adenocarcinoma of the lung. (A) Complex adenoid structure of the tumor cells. Necrosis is observed in some regions (HE × 100). (B) Solid and trabecular structures of the tumor cells (HE × 100). (C) Tumor cells form a papillary cystic structure and invade the bronchial cartilage (HE × 100). (D) The ducts of the bronchial glands are affected by tumor cells, and necrosis is observed in the lumen (HE × 100). (E) Tumor cells forming cord-like structures with fibrous hyalinized stroma and invading nerve fibers (HE × 100). (F) Tumor cells forming an eddy-like structure surrounding the nerve fibers. Most tumor cells were medium-sized and uniform in shape with oval or round clear nuclei. Nucleoli and mitotic figures can be easily observed (HE, × 200).

Microscopically, the tumor exhibited infiltrative growth close to the bronchial cartilage and bronchial glands, with a variety of histological patterns, including solid, complex adenoid, trabecular, papillary-cystic, and especially eddy-like structures surrounding the nerve fibers. The tumor stroma was fibrous, hyalinized, or mucinous. Most tumor cells were medium-sized and uniform in shape with oval or round clear nuclei. Nucleoli and mitotic figures were easily observed in some regions. Necrosis was also observed. The bronchial cartilage was found to be invaded by the tumor cells. Some ducts of the bronchial glands were also involved by the tumor tissues (Fig. 2). Tumor metastasis was observed in 1 of the 18 resected lymph nodes. Histopathological features indicated that this tumor was more atypical and aggressive than the low-grade tumors.

**Figure 3:**
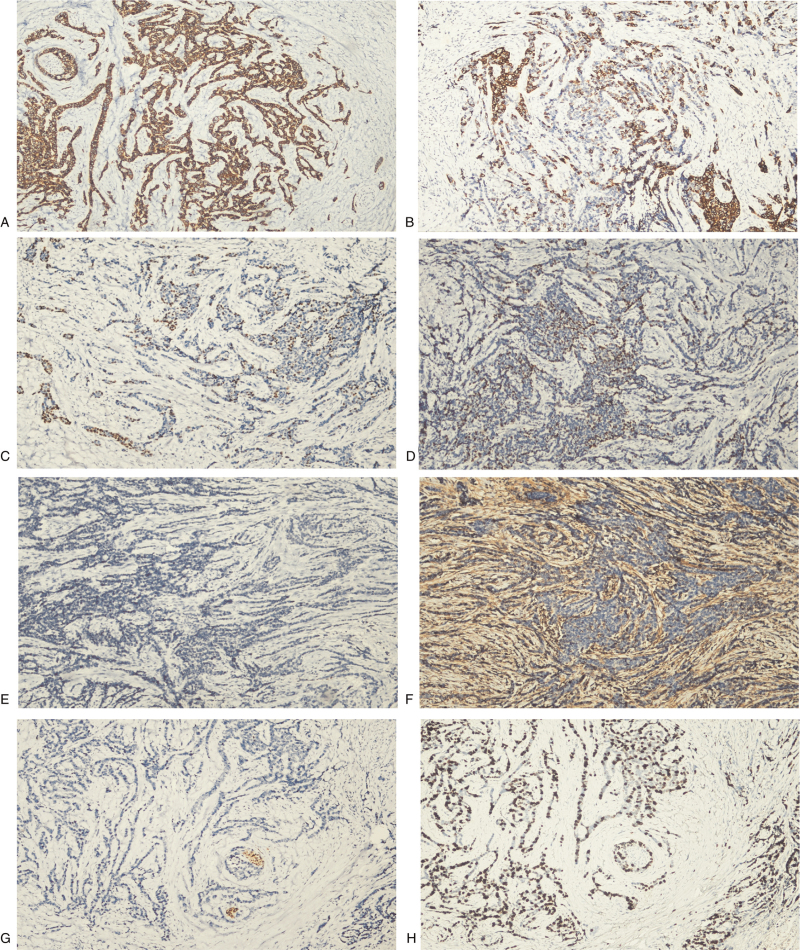
Immunohistochemistry of the polymorphous adenocarcinoma of the lung. (A) Immunohistochemical staining for CK is diffuse in the tumor cells (× 100). (B) Tumor cells are partially positive for CK5/6 (× 100). (C and D) Tumor cells are focally and scattered positive for P63 and P40 (× 100). (E) Tumor cells are negative for TTF-1 expression (×100). (F) Tumor cells are negative while stroma cells are positive for Vimentin staining (×100). (G) Tumor cells are negative while nerve fibers are positive for S-100 (×100). (H) The Ki-67 index of tumor cells is more than 40% (×100).

Immunohistochemically, the tumor cells were diffusely and strongly positive for CK. The staining for CK5/6, P63, and P40 was moderately intense and focally scattered. CD56 staining was focal. CD117 staining was scattered and moderately intense. Tumor cells were negative for vimentin, CK7, smooth muscle actin, napsin A, synaptophysin, S-100, and TTF-1. The Ki-67 index of tumor cells was more than 40% (Fig. 3).

Based on clinical information, morphological features, and immunohistochemistry results described above, the pathological diagnosis was primary PAC of the lungs (high grade), stage IIIA (T2N2M0).

## 3 Discussion

Salivary gland-type neoplasms occur at multiple organ sites in view of the basic structural homology among exocrine glands. Primary salivary gland-type tumors of the lung are rare, with several histologic subtypes; they constitute <1% of all pulmonary tumors.^[6,7]^ Primary PLGA/PAC of the lung is an extremely rare subtype of salivary gland-type tumors of the lung, which is thought to arise from the mucous glands of the bronchus. We searched primary PLGA/PAC of the lung in the PubMed database. To the best of our knowledge, including the present case, only 5 patients with primary PLGA/PAC of the lung have been reported so far. The first case of primary PLGA of the lung was reported by Lee et al^[8]^ in 2004. Clinicopathological features of the patients are summarized in Table 1. There were 4 female and 1 male patients with primary PLGA/PAC in the lung (age: 53-66 years; mean age: 57.8 years).^[8–11]^ The diameters of the tumors ranged from 1.8 to 3.5 cm (mean: 2.7 cm). The tumors had metastasized in 3 patients.

**Table 1:** Summary of primary PLGA/PAC in the lung features.

No.	Year	Sex	Age	Size (cm)	Site	Metastasis	Therapy	Outcome
1^[8]^	2004	F	56	3.5 × 3.5	2 peripheral lung lesions (1 in each lung)	Yes	Wedge resections	Alive free of disease, 9 mo
2^[9]^	2007	F	66	1.8	Right main bronchus	No	Sleeve right upper lobectomy and mediastinal lymph node dissection	Alive free of disease, 2 mo
3^[10]^	2009	F	56	3.0 × 2.5	Endobronchial	No	Resection of left upper and lower lobes	Unknown
4^[11]^	2015	F	53	3.0 × 2.0	Endobronchial	Yes	Right middle lobectomy and mediastinal lymph node dissection	Unknown
5 (present case)	2021	M	58	2.0	Bronchial lumen of the basal segment	Yes	Resection of the middle and lower lobe of the right lung, dissection of mediastinal lymph nodes	Alive free of disease, 3 mo

F = female, M = male, mo = month, PLGA/PAC = polymorphous low-grade adenocarcinoma/polymorphous adenocarcinoma.

The histological features of the primary PLGA/PAC of the lungs are similar to those of their counterparts in the salivary glands of the head and neck. PLGA/PAC of the salivary gland is an infiltrative tumor that exhibits a variety of architectural patterns, including tubular, glandular, cribriform, solid, trabecular, papillary cystic, and eddy-like structures. Perineural involvement is common.^[1,12]^ The tumor cells were small to medium in size and uniform with round or oval shape, without glandular or myoepithelial differentiation. Tumor cells were often positive for CK, CK7, P63, and S-100, but negative for other myoepithelial markers. CD117 expression was also partially positive.^[13]^ The morphological and immunohistochemical results of our patients were consistent with these features. However, the present sample showed exhibited a greater degree of cellular atypia and more mitotic figures; thus, the sample was more aggressive than low-grade PLGA. Therefore, the patient was diagnosed with PAC of the lung.

It is important to distinguish this tumor from other primary or metastatic tumors with similar histological features, such as squamous cell carcinoma, adenocarcinoma, neuroendocrine tumors, and other salivary gland-type tumors. Squamous cell carcinomas usually exhibit pronounced keratinization and intercellular bridges and is diffusely positive for P63 and P40; adenocarcinomas are positive for TTF-1 and napsin-A; and neuroendocrine tumors are positive for CD56, synaptophysin, and chromogranin.^[14]^ PLGA/PAC exhibited diverse growth patterns, without keratinization. The expression of P63 in PLGA/PAC was focal and scattered, indicating that the tumor cells had no squamous or myoepithelial differentiation.^[13]^ PLGA/PAC is negative for TTF-1, napsin-A, and neuroendocrine markers, which can help distinguish it from lung adenocarcinoma and neuroendocrine tumors.^[13,14]^ Lung metastatic PLGA/PAC and other salivary gland tumors should be ruled out based on detailed medical history and clinical examinations. Other primary salivary gland-type tumors of the lung, such as pleomorphic adenoma, adenoid cystic carcinoma (ACC), mucoepidermoid carcinoma (MEC), and epithelial-myoepithelial carcinoma, are more difficult to distinguish from PLGA/PAC because of their similar origins and histological structures.^[13,14]^ Pleomorphic adenoma is usually well-circumscribed, without normal lung tissue or perineural invasion. It is composed of variable epithelial and myoepithelial components with a mixture of patterns, and with myoepithelial cells melting into the myxoid and cartilaginous matrix. ACC and epithelial-myoepithelial carcinoma have 2 distinct types of tumor cells, with glandular epithelial and myoepithelial differentiation.^[13]^ Translocation of the *MYB* gene is also a useful diagnostic biomarker of ACC.^[15]^ MEC is an epithelial tumor composed of epithelial, intermediate, and mucous cells.^[16]^ Translocation of the *MAML2* gene is a useful diagnostic biomarker of MEC.^[17]^

Complete surgical resection of PLGA/PAC of the lungs is both curative and feasible. According to our literature review, including the present case, all 5 patients underwent surgery alone.^[8–11]^ The definite histopathological prognostic factors of PLGAs/PACs of the lung have not been fully clarified because of their rarity. Of the 5 patients, 1 patient was alive after 2 months, 1 patient was alive after 9 months, and 2 patients had no follow-up history. In our case, the patient underwent surgery alone and survived without recurrence 3 months after surgery.

In summary, we report a rare case of primary pulmonary PAC. Complete surgical resection is the treatment of choice. Careful assessment of histological features and immunohistochemistry enable efficient diagnosis.

## Acknowledgments

We would like to thank Editage (www.editage.cn) for English language editing.

## Author contributions

**Funding acquisition:** Hong-Tao Xu.

**Methodology:** Hong-Bo Xu, Jing-Ru Wang, Hong-Feng Qi, Xu-Yong Lin, Hai-Ning Zhang.

**Writing – original draft:** Hong-Bo Xu, Mai-Qing Yang, Hong-Tao Xu.

**Writing – review & editing:** Mai-Qing Yang, Hong-Tao Xu.
